# It’s OK not to be OK: Shared reflections from two PhD parents in a time of pandemic

**DOI:** 10.1111/gwao.12465

**Published:** 2020-06-23

**Authors:** Amal Abdellatif, Mark Gatto

**Affiliations:** ^1^ Accounting & Financial Management Northumbria University UK; ^2^ Leadership & Human Resource Management Northumbria University UK

**Keywords:** feminism, intersectionality, masculinities, parents, single‐parenting, vulnerability

## Abstract

Adopting an intersectional feminist lens, we explore our identities as single and co‐parents thrust into the new reality of the UK COVID‐19 lockdown. As two PhD students, we present shared reflections on our intersectional and divergent experiences of parenting and our attempts to protect our work and families during a pandemic. We reflect on the social constructions of ‘masculinities’ and ‘emphasized femininities’ as complicated influence on our roles as parents. Finally, we highlight the importance of time and self‐care as ways of managing our shared realities during this uncertain period. Through sharing reflections, we became closer friends in mutual appreciation and solidarity as we learned about each other’s struggles and vulnerabilities.

## 1 INTRODUCTION

Protecting your family is one of the most important roles you can play as a parent, but what happens when you cannot shield yourself or your loved ones from the threat of trauma (Cobham & Newnham, 2018)? These reflections provide a glimpse into the lives of two PhD parents. Amal is a second‐year PhD student (international), an associate lecturer, and a single parent to a three year old and a 13 year old. Mark is a third‐year PhD student (home), a research assistant, a co‐parent with a 14‐month‐old baby (13 months old during reflections) and his wife works in the NHS. We are both exploring gender in the workplace for our PhDs. Our shared stories of the UK COVID‐19 lockdown acted as both individual catharsis and collective empowerment. Through sharing, we both learned more about our intersectional identities and our efforts to act as protective shields for our families during this traumatic time. Importantly, we also chose to write together to expose and resist patriarchal models of gender through our divergent parental roles and converging feminist principles towards gender equity.

We present our reflective stories in three acts represented in a single day: morning, afternoon and evenings of 10 April 2020 — ‘Good Friday’ (additionally recorded as a shared time‐log exercise). This method provided a reassuring structure for us to work with, while also framing our lived experiences thematically and over a longer time period. We include snapshots of our ‘Good Friday’ to highlight how our days progressed with various points of similarity and differences. We also include reflections on our developing response to the lockdown from across a three‐week period from the start of the UK lockdown on 23 March until 8 April 2020. We intentionally shared our reflections with each other after each new entry to enrich our collective writing experience, a process which had the additional benefit of deepening our friendship and mutual admiration.

We were inspired in our collective writing approach by ‘Writing Resistance Together’ (Ahonen et al., 2020). We also drew on Grenier (2015) as a model for constructing our shared autoethnography in a quasi‐conversational form that expresses insights into our shared truths. We present our captured stories as both ordered and messy including occasional spelling and grammatical errors; a messiness that reflects our lives. By making ourselves vulnerable in this way, we hope our reflective stories can pay tribute to the canon of emancipatory feminist writing (e.g., see Cixous, Cohen, & Cohen, 1976; Haraway, 1991) that challenges how we write about ourselves and our experiences as feminists who aspire to transcend gender binaries.

## 2 MORNING: INTERSECTIONALITY AND IDENTITY

*It is not sufficient to have an experience in order to learn. Without reflecting on this experience, it may quickly be forgotten, or its learning potential lost.* (Graham Gibbs, 1988)

We share similar identities as PhD students and parents. Through our reflective diaries, we found our experiences converge around these two intersectional identities (Crenshaw, 2018). Yet, our diaries also reflect how our experiences diverge from the other identities we hold; gender, ethnicity and co‐parenting versus single‐parenting; all of which influenced our pandemic reality. We echo Rodriguez, Holvino, Fletcher, and Nkomo (2016) in moving beyond the favoured triumvirate of gender, race and class to building a more complex ontology of intersecting socio‐cognitive categories in our experiences. As we both believe in the principles of social equity, we examined and acknowledged where our identities were privileged or discriminated against in a pandemic. We feel this represents a foundational step of our feminist reflective praxis.

**7:00 a.m. —** *Trying a new strategy, waking up early to get some PhD work done, before my kids wake up.* **(Amal)**

**07:30 —** *Started PhD work on transcriptions.* **(Mark)**

*Being a parent is a full‐time job, let alone single‐parenting a teenager and a toddler, and being an international PhD student in a country away from home and family. My positionality and the intersection of my identities as single parent, PhD student, a foreigner and a woman, made me create my own worldview of the pandemic effect on life.* (Amal, 23 March)

*I’m also experiencing degrees of fatigue, because of my early starts and late finishes to squeeze in my PhD as far as I can. But these are minor trifles in comparison to what I know, others will be experiencing.* (Mark, 31 March)

**08:00 —** *Coffee in PEACE before the madness starts (first time to happen in a long time).* **(Amal)**

**08:45 —** *By the time I started to get focused, my toddler just woke up.* **(Amal)**

**07:50 —** *My son stirs and starts talking & winging intermittently (normal for him). I squeeze in a few more minutes before going to him.* **(Mark)**

*In Egypt 2011 revolution, we lived tough times. Curfews, shortage in food, unsafe streets that forced us to stay home for months. Yet, I wasn’t that scared. Maybe because I was in my country, near my parents and supported by my partner back then. Though, this past experience didn’t mitigate the shock of living a pandemic, and alone.* (Amal, 23 March)

*Typically when we think about intersectionality in Parenthood, One would consider ethnicity, socioeconomic conditions, sexual orientation, disability and other protected factors as areas of intersecting experience. For my co‐author and I, our primary intersection experience comes through our PhDs.* (Mark, 7 April)

*Being a PhD student at this time added fundamental pressures to my life as well. Since this is the reason why I left my home for, seeing how the pandemic affected life is stressful physically, psychologically and mentally.* (Amal, 23 March)

*The physical and mental battle I have discovered this week is a direct consequence of this ongoing situation. As a working parent from home with a baby, there is no option but to spend my son’s waking hours with him as entertainer, feeder and overall carer.* (Mark, 23 March)

*I find myself to be disadvantaged in the current situation. Not only do I have to worry about my study progress in this uncertain time, but also about the wellbeing of my two children and my role to support them, about my parents who are abroad, and about my residence status given this uncertain time. Yet, being aware of my positionality in relation to others is important. I see myself to be yet privileged in relation to others. Whether this includes the heart‐breaking stories of other women who are experiencing domestic abuse or those who are experiencing a job loss and financial difficulties and others who are living a trauma of losing their children, parents or partners.* (Amal, 7 April)

*I have been reflecting on my relative privilege in comparison to my co‐author. As someone who is able to draw upon the daily support of my partner, and the mother of our son. Additionally, I am a native citizen of the UK, whereas my co‐author was born and lived overseas, prior to commencing PhD and masters study in the UK. These differences in our positionality, and experiences render my parental experiences wholly different, and potentially less overwhelming than my co‐author.* (Mark, 7 April)

**09:00 —** *Breakfast and morning wash up.* **(Amal)**

**09:30 —** *Story, Play‐Doh and building blocks time with my toddler.* **(Amal)**

**08:00 —** *Help my wife pack her bag as she leaves the house for her shift at Hospital.* **(Mark)**

**09:00–09:15 —** *He plays downstairs while I tidy up a bit in the kitchen next door.* **(Mark)**

*Being an international student, and a single parent with no family members here, I was heavily reliant on others’ support. Whether this support related to childcare and schools. Or whether this support in the form of interacting or chatting with my colleagues and friends at university. Or even something as simple as taking a little break from all the pressures and responsibilities I am facing on my own by simply sitting in a café with a cup of coffee to think and reflect, while my kids are in their nursery and school.* (Amal, 4 April)

*I find myself in this position as a PhD student and husband to a key worker, my wife is a doctor in the NHS … This was my first week of being an official primary caregiver for a sustained period of three days and it has been a time of self‐discovery, love and protection … I have truly come to know him in a way that previous care just can’t replicate.* (Mark, 23 March)

**10:30 —** *Struggling to find an empty slot for grocery delivery, which means in the next few days I will have to go myself and leave my kids at home (which is very stressful with the long queues and that my toddler can’t be left alone with her brother for long).* **(Amal)**

**10:20 —** *Out for about an hour, return home at 11:20. Went to the park and returned via high street. Enjoyed listening to Sapiens by Harari in one ear and the sounds of birds and the wind through the trees in the other. He was quiet and looked around a lot at the scenery.* **(Mark)**

*My son’s school assumes I can homeschool my son all the subjects. My daughter nursery sending emails reminding of the phonics and songs to support her at home. Add to this my responsibility as a student and as tutor. Each one of those identities in itself need a full day worth work.* (Amal, 24 March)

*As a single woman in a foreign country, institutions have failed me in acknowledging, considering and appreciating how living a pandemic form my positionality and intersectionality can have an more negative impact on my life.* (Amal, 30 March)

*I have been conscious in conversation with PhD student parents that our experiences are different to other PhD students. We must protect our study time. Just as we must protect our time with our families. And these two worlds, though, naturally will cross over. Always means that we strain to separate and retain difference.* (Mark, 7 April)

*Moulding people and activities without mindfulness to individual circumstances. For example, delivering an online seminar assuming that I will be (by default) living with a partner who can help baby set the kids while teaching. Or even assuming that I will be ok with sharing the most private space, my home, my kids or even pets, with everyone. Or simply assuming that while I’m in self‐isolation, I will definitely (by default) have a neighbour/friend who can collect my medicine and drop it off at my door. Or if I got really sick I will have someone else, any other adult in my household, to contact 111 for me, although I am the only adult in my household. That was not my case.* (Amal, 30 March)

*As a parent, whose parenting experiences are still in their relative infancy, I look to my co‐author, as an experienced mentor of parenting. And someone, for whom I hold great admiration. As an extremely dedicated and resilient individual their sense of self and determination to succeed are qualities I would hope to instil in my child as a parent, and certainly qualities that inspire me as a as an aspiring academic and parent.* (Mark, 7 April)

**11:00 —** *Chatting with my son about plans to getting the school work organized, with targets, and timeframe to get it submitted.* **(Amal)**

**11:30 —** *Preparing an early lunch — reheating pasta bake with additional cooked pasta to expand the meal.* **(Mark)**

*I found reading the news, the tsunami of emails from everywhere (uni, school and nursery) to be very stressful and costing me a lot, at this time I decided to take a time off for my own mental health to be able to perform my role as the only caregiver, teacher, cook, entertainer and most importantly a friend to my kids … I decided to save my energy to what I care about most, and the reason why I wanted to pursue a PhD qualification, to make them proud and lead by example.* (Amal, 25 March)

*In the context of such an existential threat, I find it comforting to embrace the immediate love of and for my son, wife and family. Being a parent at a time like this does make you focus on what is really important and for me it has come down to my love for family and friends. Realizing my love for these people in my life has provoked one final insight, my need to protect it.* (Mark, 23 March)

*Sometimes I cry, but when I see me kids, I feel I’m lucky I have them. Every day in the quarantine, my kids and I get closer and happier. We learned how to make pizza together, we planned a daily dance session, we practice a daily gratitude by having a triple family hug, we announced Fridays to be our movie popcorn day. Together, we are trying to create a little happy place in the middle of the chaos and cope with the new life, which seems not to be as dark as I thought it would be.* (Amal, 28 March)

*The common experience that we can draw on is that of trauma and the need to protect your child act as a protective shield to your child’s emotions. For me as a parent of a 13 month old child, I am fortunate in that I can shield my child, very effectively from the ubiquitous discourse surrounding daily news bulletins Internet traffic, television, updates, etc. However, I am acutely aware that my emotional presentation with my child can inadvertently transfer feelings of anxiety, distress concern tension to my son. I have therefore opted to shield myself from the daily news cycle.* (Mark, 7 April)

*Every day I play information control, managing my impressions and facework when I interact with my children, my primary audience, at home. I try my best to be positive, even when I’m devastated by all what is going on, to lessen the ‘cabin‐fever’ effect on them. It is my responsibility to protect them and mitigate the negative impact of the new reality on their phycological, emotional and mental health.* (Amal, 2 April)

*I find myself constructing opportunities in the day to immerse myself in play with my son. This is something that serves as a twofold benefit. Firstly, I’m able to disconnect from the world around me. And to consciously participate in my son’s world oblivious, as he almost certainly is, of the ensuing pandemic. Secondly, by engaging him in play. I am also able to build greater degrees of psychological bonding with my son.* (Mark, 7 April)

## 3 AFTERNOON: MASCULINITIES, EMPHASIZED FEMININITIES AND FEMINISM

*True resistance begins with people confronting pain … and waiting to do something to change it.* (hooks, 1990, p. 229)

We found resemblance between our diary reflections around feminism and examining our femininity and masculinity in the context of COVID‐19. We reflected on Mark’s experiences of ‘re‐embodied masculinity’ (Connell, 2005) to embrace his caring role against the cultural template of ‘hegemonic masculinity’. At the same time, Amal reflected on her resistance through single‐parenting to the cultural template of ‘emphasized femininity’ (Connell & Messerschmidt, 2005). We present our non‐conformant femininities and masculinities as a challenge to the institutionalized, ‘assumed’ and regulated practices around our gender (Butler, 2004).

**13:00 —** *Cooking lunch while cleaning.* **(Amal)**

**12:00 —** *Lunch finished. He thoroughly enjoyed his pasta bake with some banana and orange segments to finish. It is very satisfying for me to see him eating the food that I’ve prepared. I feel a sense of personal gratification. I think it is very much associated with nurturing as part of my personality and the need for my food to be appreciated and to nurture my son.* **(Mark)**

*I always rebelled and used my voice, even when I was forced not to, to raise my concerns on why women should be disadvantaged in relationships, systems and societies. Why as a woman, by default, I’m expected to have a limited career ambition and should only focus on creating a ‘home’ that supports, maintains and reproduce the masculine hegemony in society and workplace. Why my only role is to be a mother, a homemaker and a caregiver? This is not assume that I don’t love and always prioritize my kids, but shouldn’t this be the case for any parent?* (Amal, 23 March)

*My personal version of masculinity has been constructed with a feminist framework. This framework of masculinity is very much designed to complement, my partner, my wife’s career aspirations. She is the primary wage earner in our relationship. She has achieved a lot in her career and will achieve more. And I want to support that, as she develops in her career. I have been studying a PhD for two years now, and that study has actually meant that I’ve been able to spend a lot of time at home, supporting childcare with my wife, but my wife has taken time off to care for our son for his first year, and has therefore taken primary care responsibility during that time. So in some ways this period of time, has provided an invaluable insight into what it will be like to be the primary caregiver for future children that we have, if we are lucky enough to do so.* (Mark, 31 March)

**13:30 —** *Realized a complete silence while I was cooking, to find out my toddler so immersed in getting her artistic skills on the walls! A moment of collapse for me!* **(Amal)**

**14:30 —** *After lunch, helped her this time to draw ON the paper rather than the walls! Also coloured Easter bunny and little eggs.* **(Amal)**

**12:55 —** *I took him up and played his wind‐up musical toy. I put him in his sleeping bag, read him a story and then put him in his cot. As usual, he was not happy about this.* **(Mark)**

*I discovered new gender dynamics through interactions between my two children. My son (the older) practising some masculine domination over his sister. For example, asking her not to talk in a certain way as she is a ‘girl’. I observed similar behaviour from my daughter towards my son. For example, seeing him wearing or playing with something that does not conform to his gender, she directly says ‘this is not for boys, this is for girls’. Here, I realized how I come out as a feminist rather than only a mother and intervene in the conversation. I try to challenge the way I was raised (and resisted) and have the conversation with them both reflecting on how it is important not to gender things or behaviours and treat one another as equal.* (Amal, 23 March)

**17:00 —** *Facetime my mom and dad, also my brother and his family.* **(Amal)**

**17:30 —** *Tea and cake in the balcony with my lovely two, texted to check on my friend, also enjoying my daughter’s talent show (that what she calls it when she sings and dances solely).* **(Amal)**

**16:15 —** *Finished calling parents. It was really nice that he got to see his grandparents. He was touching the screen quite a bit too. He started to get progressively more agitated downstairs, so I brought him upstairs to watch ‘in the night garden’ (his favourite).* **(Mark)**

**17:30 —** *Made him dinner of omelette, tomatoes, cheese and milk while watching my brother playing a Facebook live set — yoghurt to finish.* **(Mark)**

*I believe this perspective enables me to make comments or reflect upon the inversion of masculinity and primary caregiving and I hope my reflections on that process might give others some insights into the value of vulnerability and the importance of men taking ownership of the diversity of roles men can perform in the home.* (Mark, 31 March)

*I came to realize that what once was my backstage, my home, is now a place where all front stage acts, actions and interactions (‘life’ activities, tasks and institutions) are expected to take place. Whether with my consent or not, this new reality is something I resist, yet, I can’t control as an individual, I always denied access to my private life and my children. This involves things as simple as sharing their pictures on platforms like Instagram for instance. I believe this should only be done with their consent; it should not be something up to me to decide.… As a resistance strategy, I decided not to use any cameras while delivering any online sessions.* (Amal, 2 April)

*My hope is that these bizarre unexpected weeks, if not months of childcare may illuminate for many who would previously have been ignorant of these realities … It may illuminate what it actually means to be a parent, and therefore, it may crystallize a degree of sympathy, if not, empathy for the lived experiences of working parents who cannot avoid their responsibilities through outsourcing. I personally hope to learn much more about the embodied experiences of caregiving … For one, I am already nursing, a lower left back gripe that is sometimes an irritation.* (Mark, 31 March)

*One day I live as a fighter, others barely lived as a survivor … On those days, when I live as a fighter, I do my best and celebrate others, even if I don’t know them! … Tweeting congratulations to someone, who I don’t know, who just passed their viva, made me feel empowered and happy, contacting my parents, brother and sister daily, checking on my friends who live alone and sending emails to my students to show support, even when I needed this support myself. This project with Mark in itself created enthusiasm and excitement in my day. To reflect, discuss and share the experience of a pandemic with another parent, colleague and friend helped me keep up momentum. Those little things that made feel happy, energized and empowered.* (Amal, 1 April)

*My studies often feel self‐indulgent in comparison and my day of play with my son seems totally unearned. It is during this fleeting moments that I remind myself that we all have a role to play during this crisis. As my wife experiences the harsh realities of the NHS during COVID‐19, my son and I are the sanctuary of family life she can return to and recharge with before heading out again. This thought comforts me and sustains my role as a parent in these troubled times.* (Mark, 7 April)

*It is something to take pride in having a member of your family who fights as front line against COVID‐19. All NHS staff are risking their lives and the lives of their families to save others’ lives. If I am to put myself in their position, it would be very difficult to even imagine it. In doing something as simple as shopping for essentials, I come back home very worried about what I could have brought to my household and spend lots of time sanitizing groceries to avoid any harm to my vulnerable son. If I am to think that these heroes are dealing with COVID‐19 patients, working so hard to save lives, and at the end of the day they have a family at home to worry about, that would be very challenging if not traumatic. Thinking about Mark and his family, given that his wife is a doctor, it is hard to imagine or live their experiences, worries and the risks they are taking as a family to save others’ lives. If I am to put myself in their position, I feel it would be very challenging.* (Amal, 7 April)

*Having a partner working in the NHS during this crisis is a very strange experience to reflect upon. Each day, when they drive off to work, I am aware that she is heading into the notional ‘front line’ of the pandemic where the risk of acquiring the virus is greatest. I am experiencing the duality of perception that I am shielding my son from the world, while also watching my wife step into the highest risk environment. I can try to protect my son, but I am helpless to protect my wife, while she protects, and saves the lives of her patients. When I consider this in the context of historic and fictional crises, I am conscious of the masculinized responses that still seem privileged today. In contrast, I am the parent left behind to care for their son while his partner repeatedly steps ‘once more unto the breach’.* (Mark, 7 April)

## 4 EVENING — TIME PRESSURE, VULNERABILITY AND SELF‐CARE

*There ought to exist for the human being, in so far as [they are] conscious of being, a certain mode of standing opposite [their] past and [their] future, as being both this past and this future and as not being them [gender pronouns adapted].* (Sartre, 1956, p. 29)

Our experiences of time have been stretched, squeezed and snapped at various stages of this pandemic. As our working days stretched into evenings, we tended to squeeze our time with more intensity until, with fatigue, we snapped. Some experiences converged, especially our moments of vulnerability and need to take time for self‐care, while others diverged. Our need to compartmentalize our time as parents and PhD students acts as an ever‐present pressure we both battle with.

**18:15 —** *Brief news update, cleaning and tidying up the jungle!* **(Amal)**

**18:15 —** *Dinner finished & my wife calls — He perks up when he hears his mum’s voice.* **(Mark)**

**18:45 —** *Start dinner for us both while he plays in room next door — feel a bit wiped.* **(Mark)**

*Time can feel very different when you’re looking after a child. I often catch myself, checking my watch to see how much of my allocated chunk of time has passed while I’m with my child during each planned activity for that period. Examples of such chunks of time include time in our garden when it is sunny. I like to use that time to give him different types of experiences of the outdoors, which is very important to me as I remember many happy childhood experiences playing in the garden, and in family gardens.* (Mark, 8 April)

*My days are ups and downs. Living a life day‐by‐day is not easy for people who their living depends on how (and for how long) effectively they plan in advance. In being busy juggling childcare, homeschooling, domestic work, remote teaching & meetings and PhD work, I lost sight on other aspects that are fundamental to myself care. I found myself prioritizing everyone and everything and putting myself at the bottom of the list.* (Amal, 1 April)

**19:30 —** *Played PlayStation with my son, didn’t win. So decided to join my daughter in her dress up party (I never get the chance to be Elsa!).* **(Amal)**

**20:00 —** *Dinner time with my kids with classical music (Peppa Pig was still at the background as well, she never leaves the scene). That was followed by a brief family dance which my son resists daily.* **(Amal)**

**19:20 —** *His mum takes him up to put him to bed while I go to sort out dinner.* **(Mark)**

**19:45 —** *He’s asleep and we have dinner.* **(Mark)**

*I decided to squeeze a little corner in the battlefield of my everyday life to nurture my soul, mind and body. I got back to meditation to nurture my spirit closing my eyes and being aware of my surroundings (even if this included, inevitably, being aware of the noisy background of my toddler fighting with her brother). I got back to things that I stopped when I was so busy running in the maze. I started to get back to painting (even when my toddler decides to draw cute little lines over my painting). Doing this helped me create small wins to celebrate, even if imperfectly.* (Amal, 1 April)

*I have found this period of self‐isolation, to be a very unusual period of psychological trauma. It is not an immediate or jolting experience. Rather, I am becoming aware of the creeping and sloth like influence of persistent isolation and lack of social engagement on a daily basis. These slow insipid chips that form on the once solid exterior of my social day, form a surface which belies the underlying growth of worry and fear that forms my background noise on a day to day basis.* (Mark, 7 April)

*I still try to find an opportunity in the time of crisis. I’m spending as much time as I can with my kids hugging, cooking and dancing with them. Yet, as the days pass, the world lose more innocent souls and I lose the sense of time. The more I have less interest in keeping contact with the outside world.* (Amal, 4 April)

**21:00 —** *Bath time for my daughter, while I was literally shutting down and can’t move a muscle.* **(Amal)**

**22:00 —** *She is resisting to sleep, even when I have read her three fav stories.* **(Amal)**

**21:00 —** *A bit of unwinding in this time watching some comfort TV before tidy up the living room where the baby bomb has gone off!* **(Mark)**

**21:30 —** *After some general procrastination including COVID‐19 news, due to fatigue, I start work again on transcriptions.* **(Mark)**

*This is going to be a period of strain on families on individuals, and parents may feel this more than most especially those who feel the pressure to maintain working outputs, alongside maintaining high quality childcare for their children. During these coming weeks. With the best will in the world. I will not be able to replicate or even approximate the incredible experiences my son has when he is at nursery.* (Mark, 31 March)

*With the lockdown, I’m double locked. Neither receiving the childcare support, nor having the chance to take a break from the ‘tsunami’ of responsibilities bombarding me over a night. I am struggling to perform other identities with the closure of nurseries/schools as the only caregiver in my household. Especially given the disturbing picture of the outside world.* (Amal, 4 April)

**23:00 —** *Needed even 1 hour for myself, even when I really needed sleep, to think and reflect on my day before sleeping at 1:00 a.m., that included another collapse moment before sleeping.* **(Amal)**

**23:20 —** *A bit more focused after relaxed mind so do more work on transcription.* **(Mark)**

**24:00 —** *End of work, read a little of my ongoing bedtime book before I drift off.* **(Mark)**

*Started to develop new strategy to help myself cope with the new reality that seems to be lasting longer than anticipated. The only problem is that the day has 24 hours! So, the sacrifice of sleep was the only solution, since I can’t work at all while my toddler is around requiring full attention.* (Amal, 26 March)

*When I attempt to do more work in the evening I tend to find myself mentally and physically tired, until much later. This is when the occasional dragging of time during the day manifests as an inversion in the evening when time seems to flow very quickly as I tried to squeeze as much work into the end of my day as possible … doing work always feels like a race against time. It is a race that I will never win.* (Mark, 8 April)

As PhD parents, we cannot escape the ticking of time and looming deadlines; we constantly feel this pressure, even at the best of times. The lockdown has meant we cannot produce the volume, nor achieve the quality of focus and output required to meet our own perceived expectations. With each passing minute, we experience prodding fingers and shouts for attention, which wrench us away from the immersion needed to produce our best work. In such a competitive discipline as academia, where success is measured on the ‘publish or perish’ continuum, our very survival as early career academics is at the forefront of our minds.

## 5 CONCLUSION

*In the midst of every crisis, lies great opportunity.* (Albert Einstein)

*To be a complete individual, equal to man, woman has to have access to the male world as man does to the female one, access to the other; but the demands of the other are not symmetrical in the two cases.* (De Beauvoir, 2011, p. 818)

Reviewing our reflections, we have both been comforted by our two lives lived in simultaneously divergent, yet similar moments of vulnerability with our families. As we have shared reflections during these early days and weeks, we have grown closer as friends, despite the enforced distance we must observe. We have glimpsed behind the veil of our professional selves, allowed ourselves to share our precious private lives and gained something far more valuable in our mutual admiration for each other as people. As Amal has embodied the total parent from teacher to chef, carer, friend and protector, while squeezing in her studies; Mark has experienced periods of re‐embodied masculinity as transient sole primary carer and support to his wife. Our experiences are unequal, but we have both gained unplanned access to the ‘other’ as working parents, peers and friends. It is this ‘other’ that builds our case to embrace our vulnerabilities as parents towards a collective strength that could endure beyond this lockdown.

Is this a beginning to an end or an end to a new beginning? Will we get back to the life we knew? In these ambiguous uncertain times, there are plenty of unanswered questions. Even with this ambiguity, we think everyone by now already will have a long ‘To Do After the Lockdown List’. This could be something as simple as a friend’s hug, a cautious handshake, a staff kitchen gossip, a chilled drink at the pub or that long overdue haircut! Since the lockdown started, in each household, we became a huge conglomerate of organizations. We are the university, the school, the nursery, the gym, the restaurant, the library and the hairdresser. Will we see this as an ugly experience that brought all social inequalities and injustice to the surface? Or will we see it as a great opportunity for family, self‐discovery, open vulnerability, resilience, love, compassion and solidarity? Will we value one another differently, or will it be a matter of time before we get back to the ‘old’ reality of busy bees buzzing around the hive? All we do know is that this shared experience has meant more to us than we anticipated. We helped each other see the light at the end of our separate tunnels and, out of our solidarity as feminists, our friendship has blossomed.

## DECLARATION OF CONFLICTING INTERESTS

The authors declared no potential conflicts of interests with respect to the authorship and/or publication of this article.
